# For Chapped Hands

**Published:** 1888-02

**Authors:** 


### For Chapped Hands.

A cream for chapped hands, which is far superior to many of the similar advertised products, is made as follows:

Quince seed........................................ʒij

Rose water........................................Oiv

Glycerine........................................Oij

Tr. benzoin........................................fʒij

Macerate the quince seed in the rose water for twenty-four hours. Strain, and add the glycerine and tr. benzoin.—*Drug Bull.*

